# Victoria as a Mother

**Published:** 1858-02

**Authors:** 


### "VICTORIA AS A MOTHER."

It is known to the informed, that the reigning house of "*Guelph*" has, by intermarriage with too close blood relations, become scrofulous, tending to insanity, which even now has to be strongly battled against, by the avoidance of too much quiet, and by the cultivation of travel to different, distant, and new, and striking localities. But with these disadvantages, coupled with scientific medical direction, the Queen is rearing a family of eight children, all of whom, as far as we can judge, are growing up in vigorous health. Very recently, the Queen's son entered a foot-race with a mob of street boys, led the whole of them, but distributed the prize-money, which he so well won, to the laggard urchins whom he had distanced. It is not, therefore, true, that "it is healthy to go ragged, to wallow in the streets, and become familiar with mud and filth." It is equally contradictory to the sentiment, that wealth necessarily engenders effeminacy; on the contrary, it shows that wealth, under the domination of intelligent medical advisers, may be made a means of physical superiority. We hesitate not to say, that wealthy parents who have sickly children have committed a double wrong against them—the wrong of neglect or ignorance, and the still greater one of having at their command the means of health, and yet failing to make their application.

